# Stronger legacy-effects of forest fertilization on soil saprotrophic fungi than on ectomycorrhizal fungi in the first decade after clearcutting

**DOI:** 10.1093/femsec/fiag091

**Published:** 2026-08-04

**Authors:** Margaux Boeraeve, Gustaf Granath, Karina E Clemmensen, Joachim Strengbom, Björn D Lindahl

**Affiliations:** KU Leuven, Department of Biology, B-3001 Heverlee, Belgium; Uppsala University, Department of Ecology and Genetics, EBC, SE-75236 Uppsala, Sweden; Swedish University of Agricultural Sciences, Department of Forest Mycology and Plant Pathology, SE-750 07 Uppsala, Sweden; Swedish University of Agricultural Sciences, Department of Ecology, SE-750 07 Uppsala, Sweden; Swedish University of Agricultural Sciences, Department of Soil and Environment, SE-750 07 Uppsala, Sweden

**Keywords:** ectomycorrhizal fungi, saprotrophic fungi, clearcutting, forest fertilization, boreal forests

## Abstract

Intensive forest management practices, such as fertilization and clearcut harvesting, are known to severely disturb biodiversity in boreal forests, but to what extent fertilization effects are still detectable after clearcutting is currently not well understood. Here we used a space-for-time approach in clearcuts of fertilized and unfertilized forests to study the dynamics of soil fungal communities after clearcutting. We re-analysed data on fungal communities in the soil organic layer of 48 clearcuts in Sweden, spanning 1–13 years post-harvest. Clearcuts of previously fertilized forests were paired with clearcuts of unfertilized forests with similar characteristics. Ectomycorrhizal fungi disappeared after clearcutting, while saprotrophic fungi initially increased in abundance and subsequently decreased as ectomycorrhizal fungi reappeared. This effect was especially pronounced for saprotrophic agaricomycetes (*Mycena, Luellia*, and *Trechispora*) and in clearcuts of fertilized forests. We conclude that certain saprotrophic agaricomycetes respond to a transient release from Gadgil suppression by ectomycorrhizal fungi and/or an increase in substrate availability after clearcutting. Saprotrophs were particularly prolific in previously fertilized stands, in line with rapid disappearance of additionally accumulated belowground organic matter, whereas ectomycorrhizal fungal communities were little impacted by previous fertilization during the first decade after clearcutting.

## Teaser

Ectomycorrhizal fungi disappear after clearcutting, while saprotrophic agaricomycetes initially increase in abundance and decrease again as ectomycorrhizal fungi recolonize – an effect that was more pronounced in previously fertilized stands.

## Introduction

Forests in northern Europe are intensively managed to meet growing demands for forestry products and to increase profitability (Simonsen et al. 2010, Payn et al. 2015). This includes planting, soil preparation, fertilization, and clearcut harvesting. However, the intensity of forest management may conflict with other societal values of forests, related to biodiversity, carbon storage and recreation (Strengbom et al. 2018, Eggers et al. 2019, Mayer et al. 2020). While forest fertilization can increase both above- and belowground carbon stocks (Boeraeve et al. 2025a, Jörgensen et al. 2021), it is also known to affect biodiversity (Strengbom and Nordin 2008, Rodríguez et al. 2021, Jörgensen et al. 2022). Clearcutting also disturbs biodiversity (Matveinen-Huju and Koivula 2008, Nirhamo et al. 2025) but, in contrast to fertilization, reduces soil carbon stocks and increases nitrogen mineralization (Smethurst and Nambiar 1990, Olsson et al. 1996). The effect of clearcutting is especially severe in species groups that depend directly on living host trees, such as ectomycorrhizal fungi (Kohout et al. 2018, Sterkenburg et al. 2019). The disturbances caused by these silvicultural practices can persist for a long time and, thus, cause legacy effects (Franklin et al. 2000), with legacy effects of various practices potentially interacting with each other (Johnstone et al. 2016).

In temperate and boreal forests, most trees associate with ectomycorrhizal fungi (Brundrett and Tedersoo 2020). The ectomycorrhizal fungal lineages evolved multiple times from saprotrophic ancestors (Martin et al. 2016, Shah et al. 2016). While these transitions generally coincided with a loss of genes coding for lignocellulose-degrading enzymes, ectomycorrhizal fungi vary widely in the extent to which they retained capacity to decompose organic matter (Miyauchi et al. 2020). Contrary to saprotrophic fungi, ectomycorrhizal decomposers are not thought to decay organic matter to acquire carbon but rather to access nitrogen (Lindahl and Tunlid 2015), and many species are sensitive to increased inorganic nitrogen availability (Lilleskov et al. 2019). Forest fertilization experiments across Sweden have found negative effects of increased inorganic nitrogen on the occurrences of the ectomycorrhizal genera *Cortinarius* and *Piloderma* (Van Der Linde et al. 2018, Jörgensen et al. 2022), which contain species with high capacity for decomposition (Packard et al. 2025). On the other hand, species such as *Thelephora terrestris* and *Cenococcum geophilum*, which mainly access easily available nitrogen, may become more abundant after forest fertilization (Fransson et al. 2000, Edwards et al. 2004). This shift in ectomycorrhizal fungal community composition towards species with a reduced capacity to decay organic matter has been linked to fertilizer-induced increases in soil carbon stocks (Jörgensen et al. 2022).

By removing the host trees, clearcutting decimates ectomycorrhizal fungi, which subsequently must reestablish in the following rotation period. Due to differences in propagule survival, dispersal and establishment capacity, and ecological niches, ectomycorrhizal fungi vary widely in their propensity to (re)colonize regenerating forests (Jones et al. 2003, Boeraeve et al. 2018). Ectomycorrhizal fungal communities in young forest stands are mostly composed of species that readily colonize from spores, e.g. species of the genera *Suillus, Thelephora, Laccaria*, and *Paxillus* (Peay et al. 2012, Kyaschenko et al. 2017, Lindahl et al. 2026). However, local abiotic conditions also affect community assembly (Boeraeve et al. 2018), and these can be affected by fertilization during the previous rotation period (Ring et al. 2011, From et al. 2015). The increased inorganic nitrogen availability in previously fertilized stands after clearcutting can thus be expected to affect ectomycorrhizal fungal community assembly. Clearcutting can also affect saprotrophic fungi, through alterations in litter and dead root inputs, environmental conditions, and by decreased competition from ectomycorrhizal fungi. Loss of ectomycorrhizal fungi has sometimes been found to result in increased decomposition by saprotrophic fungi – a phenomenon called the ‘Gadgil effect’ (Gadgil and Gadgil 1971, Fernandez and Kennedy 2016). Clearcutting, thus, causes massive changes in soil fungal communities (Kyaschenko et al. 2017), but whether this disturbance obscures legacy effects of forest fertilization into the next rotation period is currently unknown.

In this study, we set out to examine the reestablishment of ectomycorrhizal fungal communities after clearcutting, how that relates to shifts in soil saprotrophic fungal communities, and how these dynamics are affected by fertilization during the previous rotation period. This was studied in 12 forests that were clearcut the year before sampling and 36 forests that were clearcut 4 to 13 years earlier, with half of the forests fertilized during the previous rotation period. In the same sites, we already documented that one year after harvest, the clearcuts of previously fertilized forests stored about 30% more carbon and nitrogen in the soil organic layer than clearcuts of unfertilized forests, however this difference disappeared during the following 13 years (Boeraeve et al. 2025b). The previous analysis of the soil fungal communities revealed limited differences between clearcuts of fertilized forests and of unfertilized forests (Boeraeve et al. 2025b). However, that study focused mainly on effects of fertilization during the previous rotation period relative to unfertilized conditions and included only crude linear relationships with time since clearcutting, but many of the fungal and fungus-mediated responses after clearcutting are likely to be transient (e.g. the Gadgil effect) and therefore not captured by models with a linear representation of time. More detailed temporal patterns of fungal responses were not within the scope of the previous study. Here, we performed a more time-sensitive analysis that also considered non-linear temporal responses, aiming to understand the specific fungal community dynamics and decomposer mechanisms that underpinned the disappearance of fertilizer-induced soil organic stocks during the first decade after clearcutting.

We hypothesized that ectomycorrhizal taxa disappear within the first year after clearcutting, followed by a gradual establishment of efficient spore-dispersers, such as *Suillus, Thelephora*, and *Laccaria* spp. (H1). We furthermore hypothesized that nitrogen-sensitive ectomycorrhizal taxa, such as *Cortinarius* and *Piloderma* spp., would recover more slowly in clearcuts of fertilized forests compared to unfertilized forests, while other taxa, such as *Thelephora, Tylospora*, and *Amphinema* spp., would be relatively more favored by fertilization during the previous rotation period (H2). Saprotrophic fungi, and especially saprotrophic agaricomycetes, would initially increase in abundance as the ectomycorrhizal fungi decline, and then decline when ectomycorrhizal fungi start to reestablish (H3). Finally, we hypothesized that the transient dominance of saprotrophic fungi would be more extensive in clearcuts of fertilized forests (H4). To test these hypotheses, we used a combination of community metabarcoding and qPCR-based quantification of fungal ITS2 markers, to allow for quantitative analyses of the soil fungal communities in 48 forests that were harvested through clearcutting 1–13 years before sampling, half of which were fertilized during the previous rotation period.

## Data and statistical analyses

This study is based on available data published by Boeraeve et al. (2025b), collected in the Västmanland and Uppsala county in central Sweden, in 2022–2023. This dataset comprises soil edaphic conditions, vegetation, tree regeneration, and sequence data from soil fungal communities from 12 forests that were clearcut the year before sampling and 36 forests that were clearcut four to 13 years earlier (Boeraeve et al. 2025c). The study was set up in a paired design with each of the clearcuts fertilized during the previous rotation period paired with a clearcut of an unfertilized forest with similar characteristics [soil type, site index (a measure of productivity), time since clearcutting]. The two clearcuts within a pair were separated by at most 4 km, but most were within 1 km from each other. Distances between pairs of clearcuts were at most 150 km.

All sites were managed under standard forestry practices and owned by Sveaskog AB, who provided the data necessary to select the stands. Fertilization consisted of 150 kg N ha^−1^ and was applied once (n = 20) or twice (n = 4) in the previous rotation period using the commonly used Skog-CAN, which consists of ammonium nitrate with dolomite [CaMg(CO_3_)_2_], to reduce the risk of acidification. The forest before clearcutting consisted predominantly of coniferous trees, ranging from 100% *Pinus sylvestris* to 94% *Picea abies*. No stand contained more than 4% *Betula pendula* or other deciduous trees. In all of the 4 to 13 year old clearcuts the soil was scarified (i.e. mechanical disruption of the topsoil to expose the mineral soil in order to increase seedling survival and growth), and tree seedlings (*Pinus sylvestris* and *Picea abies*, both ectomycorrhizal trees) were planted. There was also some spontaneous regeneration of the two conifers and *Betula* spp., which are also ectomycorrhizal. Average tree height increased from 0.5–1 m four years after clearcutting to 2–3 m 13 years after clearcutting, depending on the tree species (Fig. S1). In the one-year-old clearcuts, the soil was mechanically prepared in two of the six pairs, and none had yet undergone tree planting. The ground vegetation was dominated by ericoids (*Vaccinium myrtilis, V. vitis-idaea*) and grasses (*Avenella flexuosa*), with no difference between clearcuts of fertilized and unfertilized forests (Boeraeve et al. 2025b).

In each clearcut, sampling took place in three circular plots (10 m radius). In each of these plots, 25 soil cores were taken in a grid (1.5 m between grid points) and the entire organic layer of these cores was pooled into one composite sample. In total, 144 soil samples were collected from 48 clearcuts. After transport on ice to the lab, samples were weighed and homogenized in a freeze-mill. A 50 ml subsample was freeze-dried and ball-milled, and 0.25 g was used for DNA extraction. The DNA extracts were subjected to PCR-amplification and sequencing of the ITS2 region and part of the large subunit with the gITS7-TW13 primer pair on the PacBio Sequel II platform (Pacific Biosciences), and ITS2 copy numbers were quantified with the gITS7-ITS4/ITS4arch primer pair (Ihrmark et al. 2012, Sterkenburg et al. 2018, White et al. 1990) through qPCR. Amplification and sequencing biases were minimised by careful optimisation of PCR cycle numbers and by using PacBio sequencing, which has much lower amplicon length bias than e.g. the Illumina platform (Castaño et al. 2020). Sequencing data was processed through the SCATA pipeline (https://scata.mykopat.slu.se), and OTUs were identified through Species Hypothesis (SH) matching against UNITE (Nilsson et al. 2019) on the PlutoF platform (Abarenkov et al. 2010). Using the genus-level primary and secondary lifestyle according FungalTraits (Põlme et al. 2020), OTUs were assigned to ecological groups (for more details, see extended methods in the Supplement).

Given the expected dynamics in total fungal biomass after clearcutting (Moore-Kucera and Dick 2008) and the difficulty in interpreting relative abundances when total biomass varies (Props et al. 2017), we estimated absolute abundances of species and species groups based on qPCR quantification (Dannemiller et al. 2014). First, relative abundances of individual OTUs, ectomycorrhizal, saprotrophic fungal and saprotrophic agaricomycete ITS2 sequences were calculated by dividing the summed number of sequences within groups, by the total number of fungal sequences per sample (both from metabarcoding data). Second, relative abundances were multiplied by the total fungal ITS2 copy number, as determined by qPCR on a per sample basis. The resulting copy number-weighted OTU table was used in further analyses. For more background on the study sites and methods, see supplementary material and Boeraeve et al. (2025b).

The raw sequence data is available in the NCBI Sequence Read Archive under BioProject PRJNA1191207 and the copy number-weighted OTU table and metadata is available at the Swedish National Data Service (Boeraeve et al. 2025c). The dataset contained 3 379 877 sequences from 7476 fungal OTUs, the majority of which belonged to Agaricomycetes (1712 OTUs), Leotiomycetes (1367 OTUs), Eurotiomycetes (538 OTUs), Sordariomycetes (458 OTUs), and Dothideomycetes (441 OTUs) (Boeraeve et al. 2025b). Of the OTUs that could be attributed to a lifestyle, the majority were saprotrophic ascomycetes (785 OTUs), followed by root-associated ascomycetes (782 OTUs), saprotrophic agaricomycetes (610 OTUs), and ectomycorrhizal fungi (487 OTUs). Three samples had less than 40 fungal sequences (all three previously fertilized and sampled 4, 4, and 6 years after clearcutting) and were thus insufficiently deeply sequenced and omitted from further analyses. The other 141 samples contained at least 946, and on average 23 471, fungal sequences.

Statistical analyses were performed using R version 4.2.2 (R Core Team 2024). The numbers of OTUs (hereafter referred to as ‘species richness’) of ectomycorrhizal, saprotrophic fungi, and saprotrophic agaricomycetes were calculated with the *vegan* R package (Oksanen et al. 2001). The abundance and species richness of ectomycorrhizal fungi, saprotrophic fungi in total, and saprotrophic agaricomycetes, as well as the abundance of the 10 most abundant ectomycorrhizal, and 10 most abundant saprotrophic fungal genera were tested as response variables in linear mixed effects models using the *nlme* package in R (Pinheiro and Bates 2024). To test non-linear responses to time since clearcutting, we included time both as a first order and second order term in the full model using the R function *poly()*. The linear mixed effects models thus included fertilization (unfertilized/fertilized), the linear and quadratic term of time since clearcutting and their interactions as explanatory variables. Pair of clearcuts and site nested in pair were added as random factors to account for within-site sampling and dependence within pairs. Abundances (fungal ITS2 copy numbers m^−2^) were square-root transformed to meet assumptions of homoscedasticity of residuals. Statistical significance of the explanatory variables was assessed through type 2 testing using the *Anova*() function from the *car* package (Fox and Weisberg 2019). To test whether community composition of ectomycorrhizal fungi shifted with time since clearcutting, a PERMANOVA was fitted with the *adonis2* function from the vegan R package (Oksanen et al. 2001).

## Results

**Figure 1:**
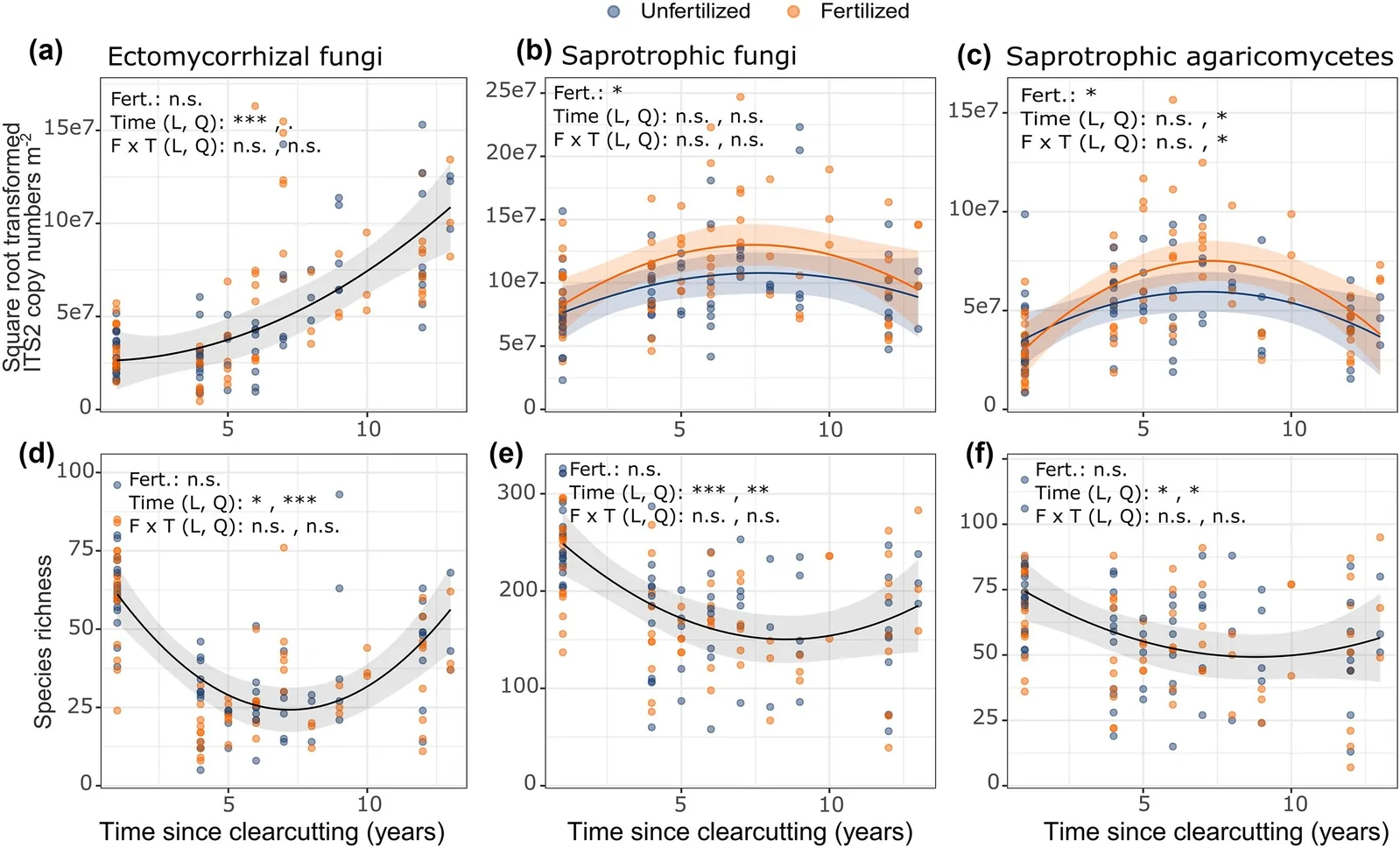
Abundance (a, b, c) and species richness (d, e, f) of ectomycorrhizal fungi (a, d), saprotrophic fungi (b, e) and saprotrophic agaricomycetes (c, f) in relation to time since clearcutting. Points indicate plot data and are coloured according to whether they come from clearcuts of previously fertilized (orange) or unfertilized (blue) forests. Predicted effects and their 95% confidence intervals from the linear mixed models for variables that had a significant effect are plotted on top of the raw data points. *P*-values for model terms are denoted as follows: n.s. (*p* > 0.1), (0.05 < *P* ≤ 0.1), * (0.01 < *P* ≤ 0.05), ** (0.001 < *P* ≤ 0.01), and *** (*p* ≤ 0.001). L and Q denote the linear and quadratic terms, respectively.

**Figure 2:**
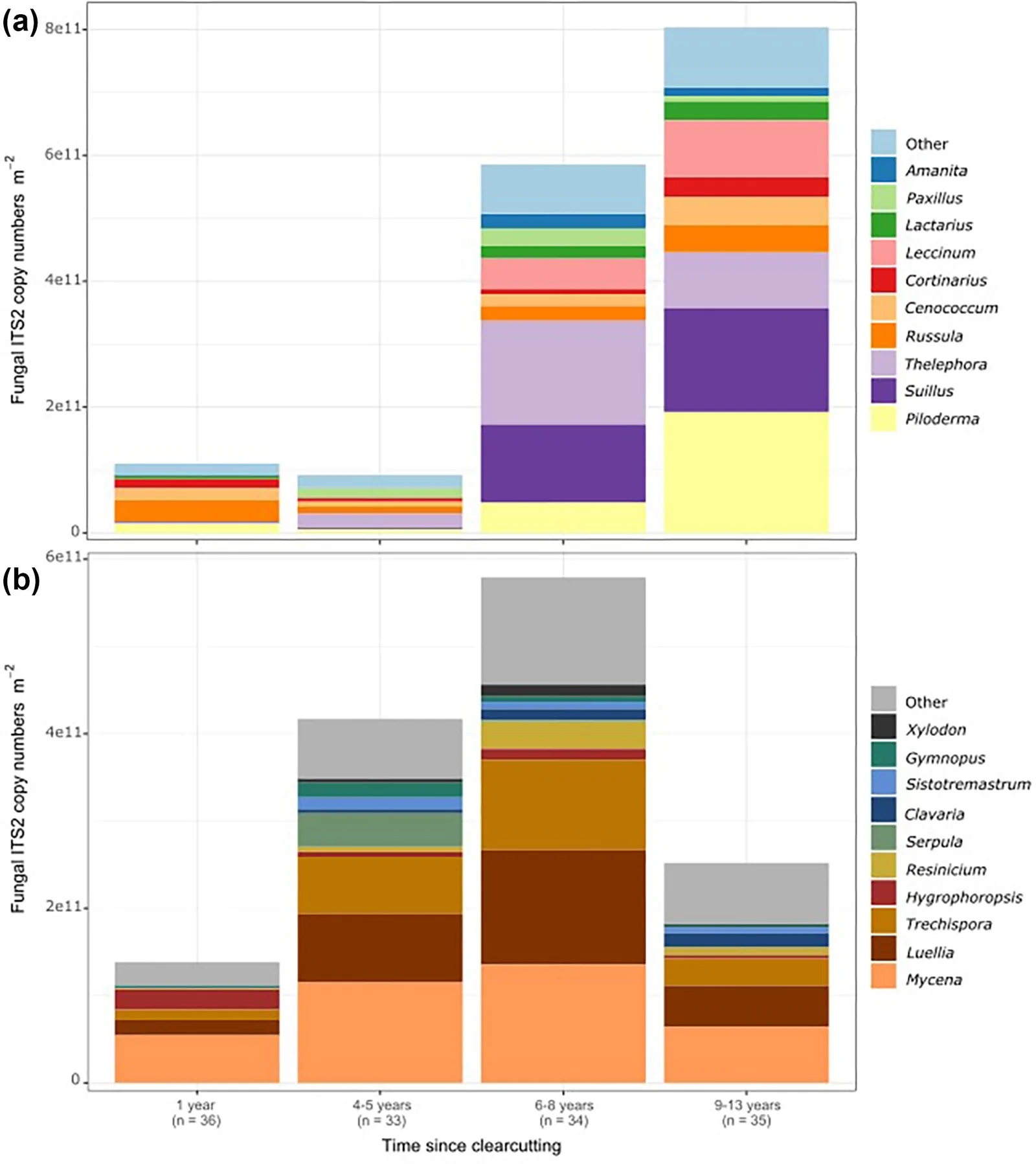
Shift in (a) ectomycorrhizal fungal and (b) saprotrophic agaricomycete abundance (measured by ITS2 copy numbers) and community composition with time since clearcutting. Bar plots indicate the average abundance of the ectomycorrhizal or saprotrophic agaricomycete genera in four age classes. Note that *Thelephora* includes former *Tomentella* (Kõljalg et al. 2024).

In line with hypothesis 1 (H1), ectomycorrhizal fungal abundance was low in the first year after clearcutting and subsequently increased significantly and linearly (Fig. 1a, Table 1). Ectomycorrhizal fungal species richness was also significantly affected by time since clearcutting, (Table 1), but with a significant negative linear and positive quadratic term (Table S1) reflecting a U-shaped relationship (Fig. 1d). Fertilization did not significantly affect ITS2 copy numbers or species richness of ectomycorrhizal fungi, and did not interact significantly with time since clearcutting (Table 1), i.e. in line with conclusions in (Boeraeve et al. 2025b). Here, however, in-depth analyses of temporal patterns revealed a clear successional shift in ectomycorrhizal fungal community composition with time since clearcutting (PERMANOVA: *R^2^* = 0.15, *F_1,88_* = 24.7, *P* = 0.001). In the first year after clearcutting, *Russula, Cenococcum*, and *Cortinarius* were the dominant genera (Fig. 2a), while 4–6 years after clearcutting, *Thelephora* was the most abundant ectomycorrhizal fungal genus, and 7–9 years after clearcutting *Suillus* and *Thelephora* were most abundant (Fig. 2a). At 10–13 years after clearcutting *Piloderma, Suillus*, and *Leccinum* were the most abundant ectomycorrhizal fungal genera (Fig. 2a).

**Table 1:** Results from the type II tests on the linear mixed models on the abundance (ITS2 copy numbers m^−2^, square-root transformed to meet assumptions of homoscedasticity of residuals) and species richness of ectomycorrhizal fungi, saprotrophic fungi, and saprotrophic agaricomycetes and on the abundance of the 10 most dominant ectomycorrhizal fungal genera and saprotrophic fungal genera. For statistical significance of the coefficients, see Figs. 1, 3, 4, and Tables S1-S3.

Response variable	Fertilization effect		Time since clearcutting effect		Fertilization x time since clearcutting interaction	
	χ²	*P*—value	χ²	*P*—value	χ²	*P*—value
Abundance EcMF	0.8	0.370	45.1	< 0.001	3.0	0.223
Species richness EcMF	1.8	0.177	41.0	< 0.001	1.3	0.512
Abundance SapF	9.2	0.002	9.1	0.011	1.9	0.392
Species richness SapF	1.3	0.259	19.1	< 0.001	0.9	0.622
Abundance SapAg	5.1	0.024	19.0	< 0.001	6.9	0.031
Species richness SapAg	0.9	0.348	7.9	0.019	2.0	0.370
**EcMF genera**						
*Piloderma*	0.1	0.771	134.9	< 0.001	12.8	0.002
*Suillus*	0.7	0.411	9.3	0.009	0.8	0.657
*Thelephora*	0.3	0.566	38.8	< 0.001	0.1	0.974
*Russula*	1.4	0.240	9.9	0.007	0.7	0.690
*Cenococcum*	0.1	0.794	16.5	< 0.001	4.3	0.114
*Cortinarius*	0.3	0.559	39.1	< 0.001	2.5	0.290
*Leccinum*	1.8	0.180	20.3	< 0.001	3.2	0.205
*Lactarius*	0.6	0.425	20.8	< 0.001	0.6	0.739
*Paxillus*	2.0	0.159	14.3	0.001	2.6	0.267
*Amanita*	2.3	0.128	1.9	0.379	2.1	0.336
**SapF genera**						
*Archaeorhizomyces*	4.0	0.045	7.4	0.024	0.4	0.836
*Penicillium*	4.6	0.032	0.7	0.696	0.1	0.955
*Mycena*	2.1	0.145	11.4	0.003	0.7	0.722
*Cladophialophora*	0.0	0.949	0.6	0.738	0.4	0.818
*Mortierella*	2.7	0.099	8.8	0.012	0.1	0.929
Hydnodontaceae (*Luellia* and *Trechispora*)	7.7	0.005	25.7	< 0.001	5.7	0.057
*Umbelopsis*	2.0	0.159	5.8	0.050	3.9	0.139
*Phialocephala*	1.9	0.161	1.3	0.513	2.8	0.247
*Solicoccozyma*	3.4	0.065	0.6	0.726	0.6	0.723

**Figure 3:**
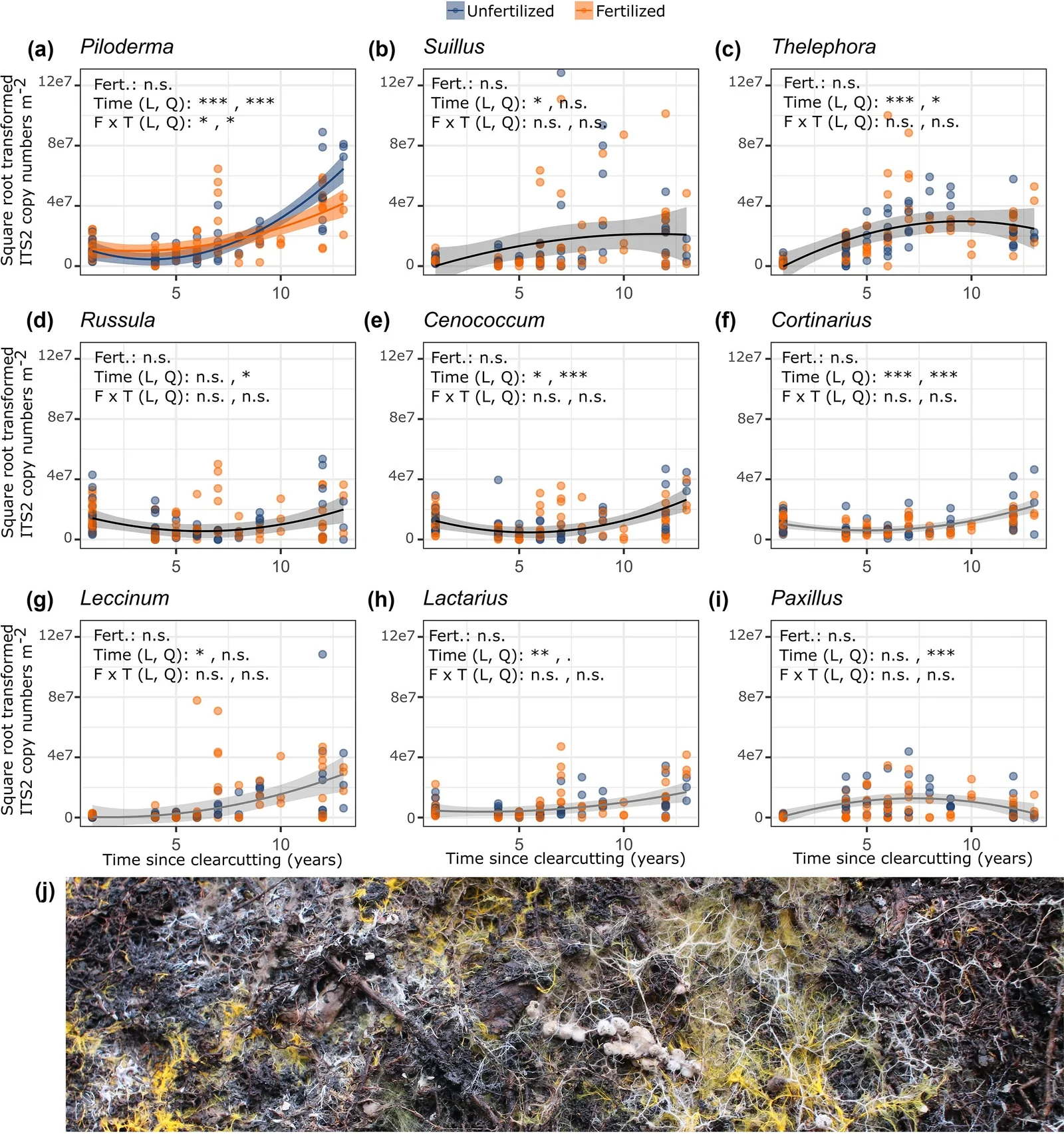
(a–i) Square root transformed abundance (ITS2 copy numbers) for nine of the 10 most abundant ectomycorrhizal genera across the gradient in time since clearcutting. Predicted effects and their 95% confidence intervals for variables that had a significant effect according to linear mixed models, are plotted on top of the raw data points. For *Piloderma*, time since clearcutting significantly interacted with fertilization during the previous rotation period (Table S3). For none of the other genera, previous fertilization had a significant effect. *Amanita* is not included in the figure since neither previous fertilization nor time since clearcutting had a significant effect. *P*-values for model terms are denoted as follows: n.s. (*P* > 0.1),. (0.05 < *P* ≤ 0.1), * (0.01 < *P* ≤ 0.05), ** (0.001 < *P* ≤ 0.01), and *** (*P* ≤ 0.001). L and Q denote the linear and quadratic terms, respectively.(j) Ectomycorrhizal root tips (*Suillus* cf. *variegatus*) and ectomycorrhizal fungal mycelia, including *Piloderma fallax* (yellow mycelia). © Margaux Boeraeve

In support of our second hypothesis (H2), the significant increase in abundance of *Piloderma* with time since clearcutting was hampered by fertilization during the previous rotation period, as evident from a negative fertilization x time interaction (Fig. 3a, Table 1, Table S3). However, this interaction was mostly driven by the oldest pair of clearcuts – a model excluding this pair did not detect a significant interaction between previous fertilization and time since clearcutting on the abundance of *Piloderma* (*χ²* = 3.3, *P* = 0.188). Reestablishment and abundance of the other abundant ectomycorrhizal genera, including *Cortinarius*, were not affected by fertilization (i.e. no significant effects of fertilization or fertilization x time). The abundance of *Suillus* and *Leccinum* increased with time since clearcutting, with a significant positive linear term, but a non-significant quadratic term (Fig. 3b and g, Table S3). However, due to the square root transformation to avoid heteroscedasticity this does not necessarily mean that the increase with time since clearcutting was linear, although models on the untransformed data did give similar outcomes (results not shown). The models on *Russula, Cenococcum, Cortinarius*, and *Lactarius* included both a statistically significant positive linear and positive quadratic term of time since clearcutting (Fig. 3d–f and h, Table S3), indicating accelerating establishment. In contrast, the models of *Thelephora* [including former *Tomentella* (Kõljalg et al. 2024)] and *Paxillus* included a statistically significant positive linear term and negative quadratic term, reflecting hump-shaped dynamics (Fig. 3c and i, Table S2). *Amanita* was neither significantly correlated with previous fertilization, nor with time since clearcutting (Table 1).

**Figure 4:**
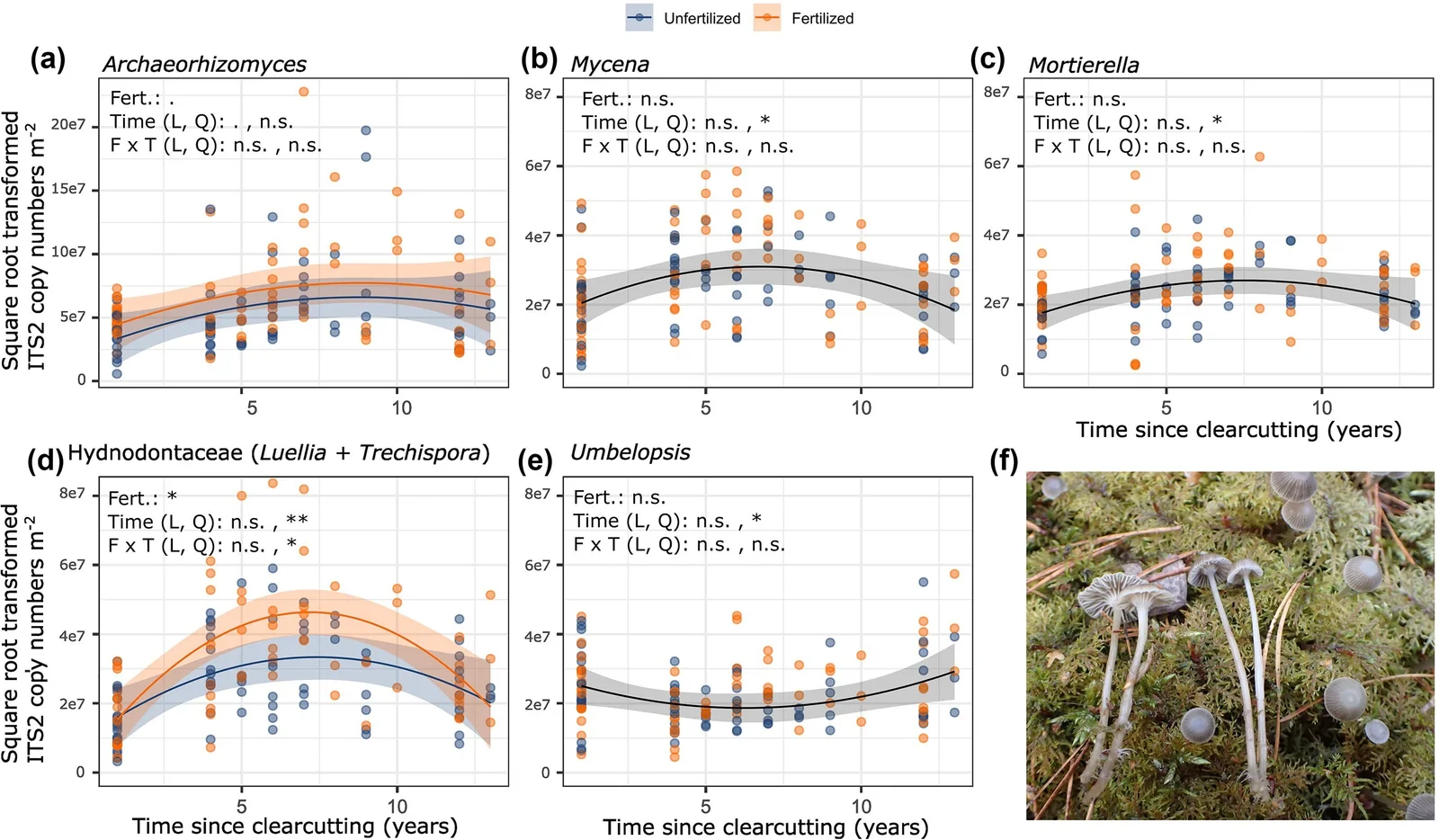
(a–e) Square root transformed abundance (ITS2 copy numbers) for 6 of the 10 most abundant saprotrophic genera in relation to time since clearcutting. For the other four genera (*Penicillium, Cladophialophora, Phialocephala*, and *Solicoccozyma*) time since clearcutting did not significantly affect abundance (Table S3), so they are not shown here. Predicted effects and their 95% confidence intervals for variables that significantly affected abundance plotted on top of the raw data points. Due to their relatedness, similar ecology and similar model outcomes, *Trechispora* and *Luellia* have been merged at the family level, Hydnodontaceae (no other genera belonging to that family were present). *P*-values for model terms are denoted as follows: n.s. (*P* > 0.1), (0.05 < *P* ≤ 0.1), * (0.01 < *P* ≤ 0.05), ** (0.001 < *P* ≤ 0.01), and *** (*P* ≤ 0.001). L and Q denote the linear and quadratic terms, respectively. (f) *Mycena vulgaris*, a common litter saprotroph in coniferous forests © Margaux Boeraeve.

The abundance of saprotrophic fungi was significantly higher in clearcuts of fertilized forests and was significantly affected by time since clearcutting, with a negative quadratic term, reflecting a hump-shaped relationship (Fig. 1b, Table 1), which supports our third hypothesis (H3). Fertilization did not significantly interact with time since clearcutting (Table 1). Similarly, abundance of saprotrophic agaricomycetes showed a positive fertilization effect (as previously reported in Boeraeve et al. 2025b), as well as a significant, negative quadratic term of time since clearcutting, reflecting a hump-shaped relationship (Fig. 1c, Table S1). Moreover, a significant fertilization x time interaction implied stronger time dynamics in clearcuts of fertilized forests for saprotrophic agaricomycetes (Fig. 1c, Table 1), in line with our fourth hypothesis (H4). The models on species richness of all saprotrophic fungi and of saprotrophic agaricomycetes included significant negative linear and positive quadratic terms of time since clearcutting, reflecting U-shaped relationships, but no significant effect of fertilization or the interaction (Fig. 1e and f, Table 1). The three agaricomycete genera (*Mycena, Luellia*, and *Trechispora*) had a hump-shaped relationship with time since clearcutting (Figs. 2b and 4) , as indicated by the significant negative quadratic term (Table S1). For *Luellia* and *Trechispora*, which were merged at the family-level, Hydnodontaceae, due to their relatedness, similar ecology and similar model outcomes (Fig. S2), the interaction between fertilization and time since clearcutting was significant (Table 1). *Umbelopsis* had a U-shaped relationship with time since clearcutting (Fig. 4), as also indicated by the significant, positive quadratic term (Table S1). *Archaeorhizomyces, Penicillium*, and *Solicoccozyma* experienced a (marginally) significant positive effect of fertilization during the previous rotation period (Table 1, Fig. 4). Finally, for *Cladophialophora* and *Phialocephala*, none of the variables included in the models were found to significantly affect their abundance (Table 1).

## Discussion

As expected, clearcutting induced large changes in soil fungal communities, with dynamic successional patterns being evident within the relatively short time span of 13 years after harvest. While ectomycorrhizal fungi disappeared after clearcutting, saprotrophic fungi increased in abundance. Especially saprotrophic agaricomycetes like *Mycena, Luellia*, and *Trechispora* increased steeply in abundance after clearcutting, followed by a decrease in abundance as ectomycorrhizal fungi recolonized. This suggests a short-term competitive release, reflecting reduced suppression by ectomycorrhizal fungi (i.e. a Gadgil effect), and a re-established competitive balance as trees and ectomycorrhizal fungi returned. This effect was even more pronounced in clearcuts of fertilized forests, suggesting that saprotrophic agaricomycetes may be responsible for the rapid disappearance of fertilizer-induced soil carbon stocks in these clearcuts, although the proliferation of these saprotrophs was not reflected in significantly elevated soil respiration (Boeraeve et al. 2025b). Despite the strong disturbance caused by clearcutting, legacy effects of fertilization were still detectable in the next rotation period, especially in the Hydnodontaceae family (*Luellia* and *Trechispora*).

In line with our first hypothesis (H1), ectomycorrhizal fungal abundance was low in the first year after clearcutting and subsequently increased. On the other hand, ectomycorrhizal fungal species richness initially decreased and later increased. In the first year after clearcutting, ectomycorrhizal fungal communities were also dominated by genera typically found in mature forests (*Russula, Cortinarius*) (Lindahl et al. 2026), suggesting that the (little) ectomycorrhizal DNA sampled in the first year after clearcutting came from dead or dying mycelia. Similarly, previous studies have found that ectomycorrhizal root tips can persist for months after clearcutting or other root disturbances, although with large differences among species (Harvey et al. 1980, Kohout et al. 2021, Kernaghan et al. 2025). Especially *Cenococcum* is known to persist for a long time, most likely due to its melanized hyphae (Fernandez et al. 2013).

Ectomycorrhizal fungal communities of 4- to 13-year-old clearcuts were dominated by early-successional genera that can readily colonize seedlings via spores, such as *Thelephora, Paxillus*, and *Suillus* (Birraux and Fries 1981, Peay et al. 2012). Remarkably, some of these early-successional ectomycorrhizal taxa (*Thelephora, Paxillus*) showed a hump-shaped relationship with time since clearcutting and levelled off, or even declined, in abundance towards the end of the 13 year period. This has been reported before for *Thelephora terrestris*, which is a common species in tree nurseries (Menkis et al. 2016) and often decreases in abundance with increasing stand age as succession proceeds (Varenius et al. 2017, Lindahl et al. 2026). This decrease seems to happen before the ectomycorrhizal biomass production peaks, which occurs in 10 to 30 year old forests at the time when the canopy is closing (Wallander et al. 2010). It thus seems like these early-successional taxa experience competitive exclusion by other ectomycorrhizal taxa that colonize the young forest stand (Kennedy 2010). This could be due to a competition-colonization trade-off (Smith et al. 2018), or because the optimal conditions for their growth disappear as the new forest establishes. Both *Thelephora* and *Paxillus* are considered to be ‘absorber’ taxa that have rapid mycelial proliferation and thrive in forests with a high supply of soluble nitrogen (Jörgensen et al. 2025) – conditions that likely occur the first years after clearcutting when there is a high turn-over of the organic nitrogen stocks and reduced tree nutrient uptake.

Fertilization during the previous rotation period had limited effects on the reestablishment of ectomycorrhizal fungi. Of the 10 most abundant genera, *Piloderma* was the only genus with a significant negative interaction between time since clearcutting and fertilization. However, the effect was driven by the oldest pair of clearcuts, where the difference between the previously fertilized and unfertilized clearcut was most pronounced. Other studies in Europe have also found *Piloderma* to be sensitive to increased inorganic nitrogen availability (Van Der Linde et al. 2018, Almeida et al. 2022, Jörgensen et al. 2024), but the opposite has been found as well in other parts of the world (Karst et al. 2021). Overall, carry-over effects of forest fertilization were mostly visible among the saprotrophic fungi, which were more abundant in clearcuts of fertilized forests. Of the 10 most abundant saprotrophic genera, *Archaeorhizomyces, Penicillium, Trechispora* and *Luellia* were significantly more abundant in clearcuts of fertilized forests, while *Mortierella* and *Solicoccozyma* also tended to be more abundant in clearcuts of fertilized forests. Previous studies have found a positive effect of fertilization on the abundance of *Penicillium* and their enzymatic activities (Arnebrant et al. 1990, Jiang et al. 2014).

Saprotrophic fungi initially increased in abundance after clearcutting, and subsequently decreased. This hump-shaped relation between abundance and time since clearcutting was especially pronounced in the saprotrophic agaricomycetes, predominantly represented by the genera *Mycena, Luellia*, and *Trechispora*. This is in line with previous studies that have found clearcutting to favour saprotrophic fungi (Kyaschenko et al. 2017, Parladé et al. 2019). However, the species richness of saprotrophic fungi decreased in the first seven years after clearcutting after which it appeared to stabilize or even increase again, indicating that the changes induced by clearcutting had negative effects on at least some saprotrophic taxa. This raises the question whether clearcutting could have long-lasting effects on the diversity of soil saprotrophic fungi; an ecological group that is severely underrepresented in studies addressing clearcutting effects on forest biodiversity (Nirhamo et al. 2025). It is possible that the proliferation of some of the dominant saprotrophic taxa led to a reduction of less competitive saprotrophs. Another possibility is that some of the taxa that were lost were inaccurately classified as saprotrophs due to the genus-level classification of the lifestyles. Of the most abundant saprotrophic genera – *Umbelopsis –* was the only that initially declined after clearcutting, but this genus also contains many root endophytes (Hoff et al. 2004), some of which have been shown to be sensitive to clearcutting (Summerbell 2005). Several other genera that were classified as saprotrophs, based on the primary lifestyle according to FungalTraits (Põlme et al. 2020), are known to also include root-associated taxa, e.g. *Mycena, Archaeorhizomyces*, and *Phialocephala* (Grünig et al. 2008, Harder et al. 2023, Thoen et al. 2026). However, these did not decline after clearcutting, potentially due to a shift from endophytism to saprotrophy in the tree roots (Kohout et al. 2018) or because of association with the ground vegetation. Intra-genus (and intra-species) variation in lifestyle constitutes an important limitation to the use of databases such as FungalTraits (Põlme et al. 2020) and should, thus, always be taken into account when interpreting metabarcoding results.

The saprotrophic taxa that increased after clearcutting later declined, coinciding with the increase in ectomycorrhizal fungal abundance, presumably as competitive suppression was reestablished when the ectomycorrhizal fungi colonised again. Previous studies have found that elimination of ectomycorrhizal fungi by trenching increases decomposition in the upper litter layer (Gadgil and Gadgil 1975, Fisher and Gosz 1986), due to alleviation of saprotrophs from nitrogen limitation (Sterkenburg et al. 2018). To decompose the nitrogen-poor fresh litter, saprotrophic fungi can redistribute nitrogen from older, more decomposed litter (Boberg et al. 2014), but are often outcompeted by ectomycorrhizal fungi at this depth (Lindahl et al. 2007, Bödeker et al. 2016). Alternatively, the increase in saprotrophic fungal abundance could be due to a temporary increase in litter and dead root inputs, although previous studies have found limited differences in soil fungal or litter-degrading fungal communities between sites with stem-only harvesting and whole-tree harvesting (Allmér et al. 2009, Hartmann et al. 2012). For saprotrophic agaricomycetes, the transient proliferation after clearcutting was further stimulated by fertilization. In clearcuts of fertilized forests, their abundance showed a stronger increase after clearcutting but was reduced to the same level as in clearcuts of unfertilized forests by the end of the study period. This further supports a potential role of increased nitrogen availability underpinning the Gadgil effect in nitrogen-limited systems, such as boreal forests. The stronger increase in abundance of these potent decomposers could also have contributed to the rapid disappearance of the difference in soil carbon stocks between clearcuts of fertilized and unfertilized forests (Boeraeve et al. 2025b). Furthermore, the proliferation of a group of potent saprotrophic decomposers after clear-cutting could potentially explain the lower carbon storage in the organic horizons of secondary (previously clearcut) forests compared to old-growth forests (Pascual et al. 2026).

Several limitations should be addressed. First, variability in copy number per genome, nuclear density, AT/GC content and length of ITS2, combined with amplification bias (Kauserud 2023), means that ITS2 copy numbers quantified through qPCR are not an exact measure of biomass. While the use of PacBio Sequel II ensures low sequencing bias (Castaño et al. 2020) and high-quality sequences, the sequencing data is probably still not a perfect representation of fungal community composition. Here, we combined two imperfect datasets (qPCR-based ITS2 copy numbers and PacBio Sequel II sequencing data) into one dataset to do quantitative analyses, thus potentially amplifying error rates. As variation in ITS copy number per genome is increasingly divergent at higher phylogenetic levels (Lofgren et al. 2019), especially affecting groupings that include different orders or phyla (e.g. ectomycorrhizal fungi, saprotrophic fungi), observed changes could be caused by changes in the relative abundance of these orders or phyla within ecological groups. Changes in abundance of single genera based on ITS2 copy numbers, however, should be quite representative of changes in biomass of that fungal genus, as the bias can be expected to be consistent across samples. Second, as this study was executed in forests under standard forestry practices, we cannot exclude that some of the effects we found are (partly) due to other forestry practices than clearcutting, e.g. soil scarification or tree planting. In a similar study in two fertilization field experiments in Sweden, Strömgren et al. (2026) found that in one of the two field experiments, there was an effect of fertilization during the previous rotation period on ectomycorrhizal fungal diversity in plots without soil scarification, while there was no difference in plots with soil scarification. So, it is possible that the absence of a legacy effect on ectomycorrhizal fungal communities in our study is not due to the clearcutting itself but due to other standard practices in this type of rotational forest management. It is, furthermore, possible that tree planting has affected the ectomycorrhizal fungal communities and especially the abundance of *Thelephora terrestris*, which is a common species in tree nurseries (Menkis et al. 2016).

## Conclusion

Clearcutting represents a strong disturbance, yet some legacy effects from forest fertilization during the previous rotation period remained. Contrary to our hypothesis, pre-harvest fertilization barely affected post-harvest ectomycorrhizal fungal community assembly, but it did affect the abundance of several saprotrophic genera. This group of fungi had a hump-shaped response in abundance to time since clearcutting, probably indicating a temporary release from Gadgil suppression by ectomycorrhizal fungi. In particular, growth of *Luellia* and *Trechispora* species (Agaricomycetes) was stimulated by previous fertilization, which may explain the rapid disappearance of fertilizer-induced organic matter stores in the organic layer (Boeraeve et al. 2025b). Clearcutting decimated ectomycorrhizal fungi, followed by a rapid community succession, in which nitrophilic ectomycorrhizal taxa like *Paxillus* and *Thelephora* displayed transient proliferation during the decade after clear-cutting.

## Supplementary Material

fiag091_Supplemental_File

## Data Availability

Raw sequence data was submitted to the NCBI Sequence Read Archive under BioProject PRJNA1191207. Data used in the statistical analyses were submitted to the Swedish National Data service (snd.se) and are available under https://doi.org/10.5878/vfre-f585 (Boeraeve et al. 2025c)
