# Supplementary material for: Stronger legacy-effects of forest fertilization on soil saprotrophic fungi than on ectomycorrhizal fungi in the first decade after clearcutting
Source: FEMS Microbiol Ecol. 2026 Aug 4;102(9):fiag091. doi: 10.1093/femsec/fiag091 (PMC13492344; doi:10.1093/femsec/fiag091)
Supplement: Supplementary file 1 [file fiag091_supplemental_file.pdf]

Supporting Information

1. Extended methodology

Sampling took place in September 2022 (four to 13 year old clearcuts) and August 2023 (one year old clearcuts). In each clearcut, three circular plots with a radius of 10 m were delineated in which soil samples were collected. Plots were situated away from retention trees. Across the plot, 25 soil cores were taken in a five-by-five grid pattern, the mineral soil was removed and the organic layer pooled into one sample per plot. The soil samples were transported on ice and stored at -20° C. Soil samples were weighed and homogenised in a freeze-mill. A 5 g subsample was shaken in 25 mL of deionised water for 10 min at 650 rpm and left to equilibrate for 15 min, after which the pH was determined with a PHM93 pH meter (Radiometer, Copenhagen). From each of the homogenized soil samples, a subsample was taken, weighed, freeze-dried and weighed again to determine % dry weight. The freeze-dried subsamples were also analysed for carbon and nitrogen content on a combustion elemental analyser (TruMac CN; LECO, St. Joseph, MI, USA) and ball milled for DNA extraction.

DNA was extracted from 0.25 g of the freeze-dried and ball milled soil with the Nucleospin Soil kit (Machery-Nagel) according to the manufacturer's instructions. DNA extracts were diluted to 1 ng μL^{-1} and used in PCR, where an approximately 1000 bp long fragment including the ITS2 region and part of the large subunit was amplified using the gITS7-TW13 primer pair (Ihrmark et al., 2012; Tedersoo et al., 2018). The 50 µL PCR reactions contained 0.5 µM forward primer, 0.3 µM reverse primer, 1.25 U DreamTaq polymerase, 0.2 mM dNTPs, 1.5 µL $MgCl_2$ and 25 ng template DNA. PCR conditions included an initial denaturation step of 5 min at 94° C, followed by 21-25 cycles of 30 s at 94° C, 30 s 56° C and 1 min at 72° C and a final elongation step of 8 min at 72° C. PCRs were done in triplicates, which were pooled per sample and cleaned with Agencourt AMPure XP beads (Beckman Coulter). After measuring the concentration with Qubit, the amplicons were equimolarly pooled and the pooled sample was cleaned with the E.Z.N.A. Cycle Pure Kit (Omega Bio-Tek). The quality and quantity of the final pool were assessed using Bioanalyzer (Agilent tech). Finally, the amplicon pool was sequenced on the PacBio Sequel II platform (Pacific Biosciences) at SciLifeLab NGI (Uppsala, Sweden). Samples from the two sampling campaigns (in September 2022 and in August 2023) constituted two separate amplicon pools that were sequenced separately but data was processed together. Sequence data was submitted to the NCBI Sequence Read Archive under BioProject PRJNA1191207.

Sequence data underwent quality filtering and OTU clustering through the SCATA bioinformatics pipeline (https://scata.mykopat.slu.se). Sequences containing both primer and identification tag sequences, with a minimum length of 200 bp (without primers and tags), average quality > 20 and single base quality > 3 were used in single-linkage clustering at a 98.75 % sequence similarity threshold. This sequence similarity threshold was chosen after clustering at four different similarity thresholds and

comparing the taxonomic identifications. The optimal threshold, with the greatest correspondence between OTUs and species, turned out to be 98.75 %. OTUs were identified through the Species Hypothesis (SH) matching service against the UNITE database (Nilsson et al., 2019), implemented in the PlutoF platform (Abarenkov et al., 2010). Identifications were double-checked by BLASTing the OTU reference sequence against NCBI Genbank. The identifications were subsequently used in combination with the FungalTraits database (Põlme et al., 2020) to attribute OTUs to the following ecological groups: ectomycorrhizal, saprotrophic ascomycetes (Ascomycota that were assigned primary lifestyle soil, litter, wood or unspecified saprotroph in FungalTraits), saprotrophic agaricomycetes (Agaricomycetes that were assigned primary lifestyle soil, litter, wood or unspecified saprotroph in FungalTraits), root-associated ascomycetes (Ascomycota that were assigned primary or secondary lifestyle root endophyte, dark septate endophyte or ericoid mycorrhizal fungus or with an unknown primary or secondary lifestyle but the SH had been found in root samples according to the UNITE database), other saprotrophs (taxa with primary lifestyle soil, litter, wood or unspecified saprotroph that were not Ascomycota or Agaricomycetes), plant pathogens, mycoparasites, other or unknown. The saprotrophic ascomycetes, saprotrophic agaricomycetes and the other saprotrophs were also put together in one group of saprotrophic fungi for further analyses.

From the DNA extracts, ITS2 copy numbers were quantified using a SYBR green-based quantitative real-time PCR (qPCR) with the gITS7-ITS4/ITS4arch primer pair (Ihrmark et al., 2012; Sterkenburg et al., 2018; White et al., 1990) on a CFX Connect Real-Time System (Bio-Rad). First, all samples were tested for inhibition by running reactions spiked with pGEM plasmid (Promega, WI, USA) and plasmid-specific primers (M13F and M13R). Then, reactions were run in 18 µL volumes consisting of iQ SYBR Green Supermix, nuclease-free water, 0.5 µM forward primer gITS7, 0.3 µM reverse primer ITS4, 0.1 µM reverse primer ITS4arch, 1 µg/µL bovine serum albumin and 0.5 ng template DNA. Reactions were run in duplicate, in two separate runs, and contained a serial dilution of a standard to produce a standard curve. The results were converted to fungal ITS2 copy numbers per unit area (copies/m^{-2}). Ectomycorrhizal and saprotrophic fungal ITS2 copy numbers and ITS2 copy numbers of individual OTUs were calculated by dividing the summed number of sequences from all ectomycorrhizal, saprotrophic fungi, or from single OTUs, by the total number of fungal sequences (both from metabarcoding data) and then multiplied by the fungal ITS2 copy numbers, as determined by qPCR, for that sample. The resulting, copy number-weighted OTU table was used in further analyses.

2. Supplemental Figures

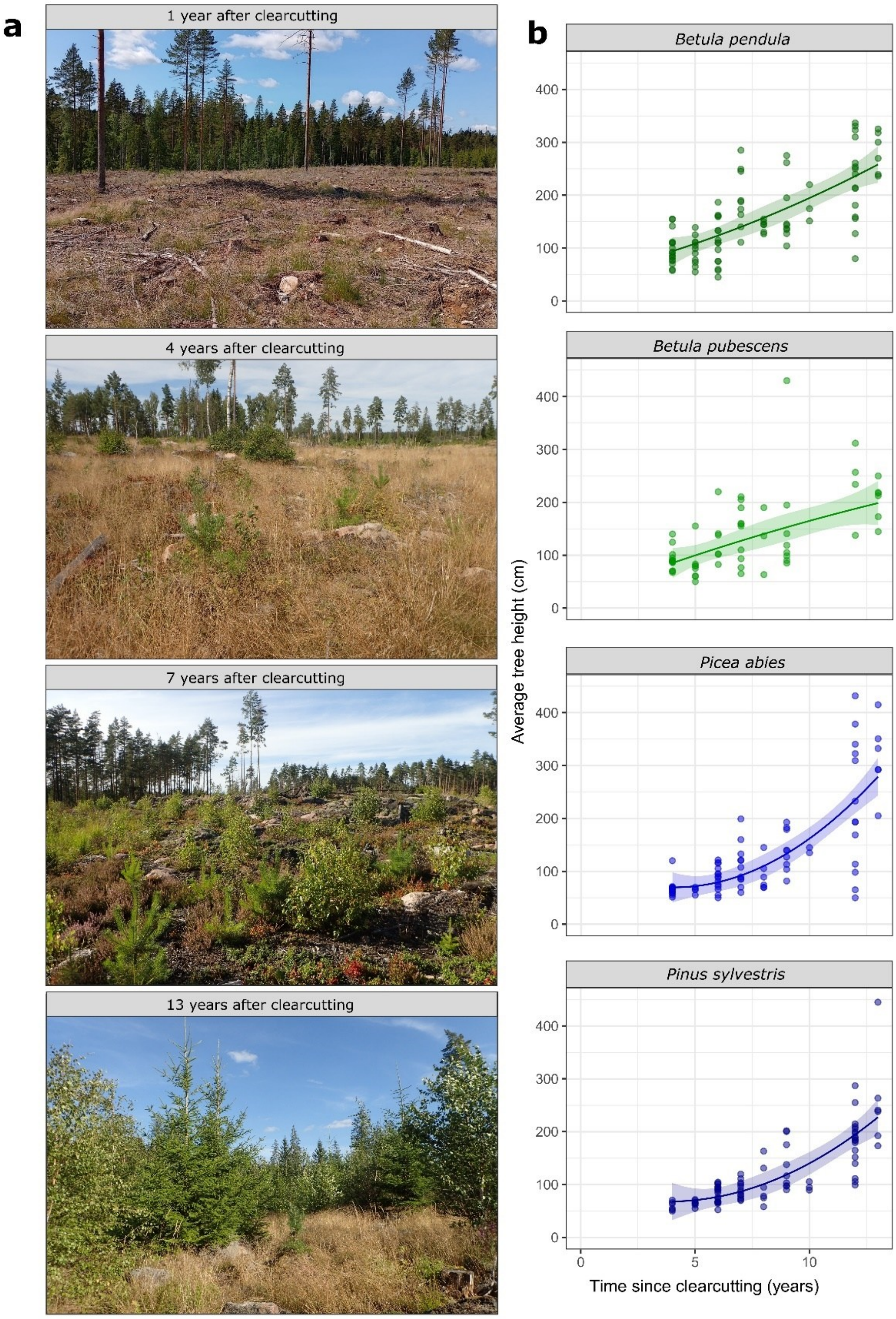

Fig. S1 (a) Pictures of four of the sampled clearcuts. **(b)** Average tree height for the four dominant tree species (two naturally regenerated: *Betula pendula* and *B. pubescens,* and two planted: *Picea abies* and *Pinus sylvestris*). The predicted effect and its 95% confidence intervals from a linear mixed model including species in interaction with time since clearcutting (both linear and quadratic term) is plotted on top of the points indicating plot-averages per species. Previous fertilization did not significantly affect average tree height.

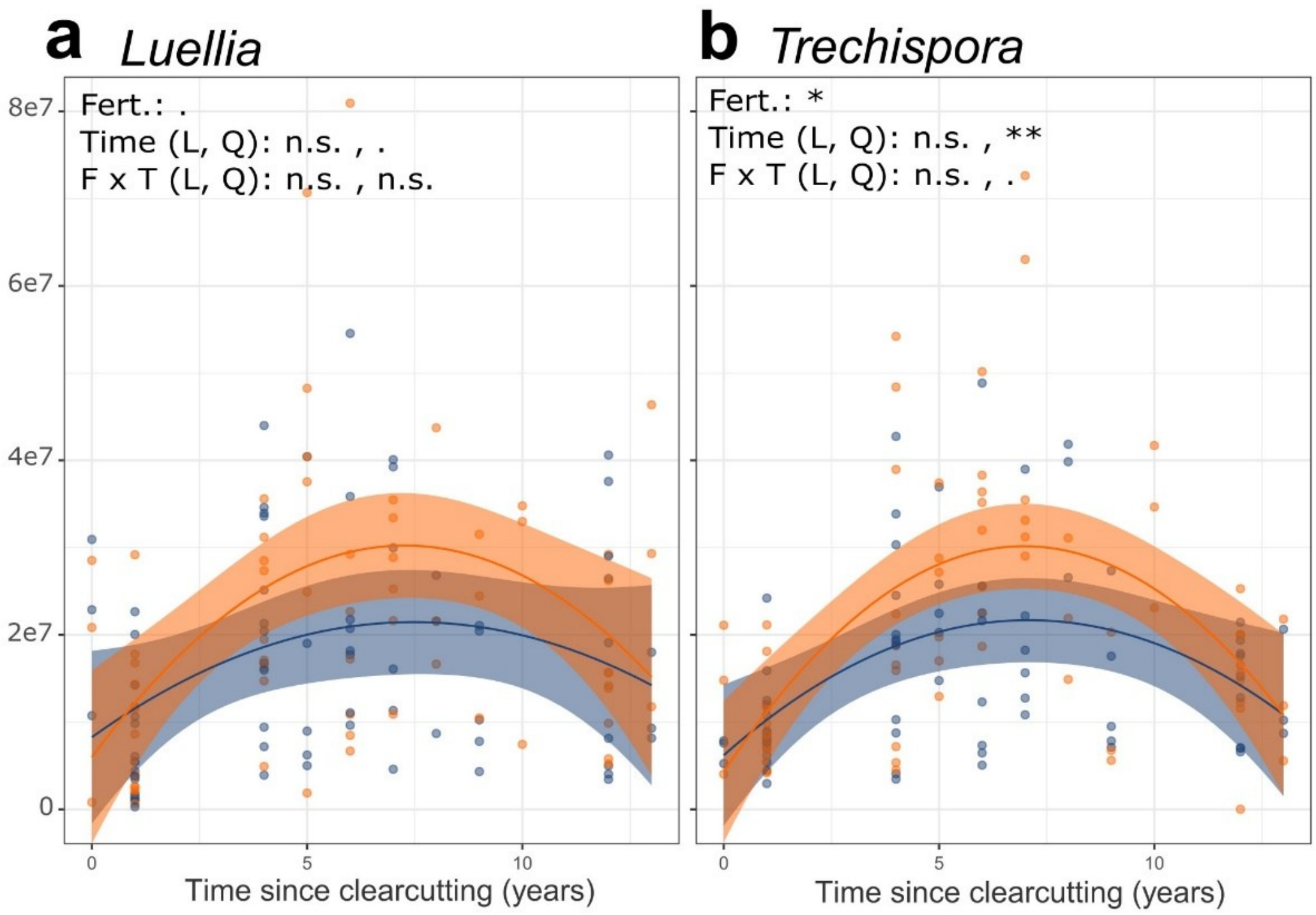

Fig. S2 Fungal ITS2 copy numbers in relation to time since clearcutting for the genera (a) *Luellia* and (b) *Trechispora*, which were merged at the family level in the main analyses because of their similar ecology, relatedness and similar model outcomes. Points indicate plot data and are coloured according to whether they come from clearcuts of previously fertilized (orange) or unfertilized (blue) forests. Predicted effects with their 95% confidence intervals from the linear mixed models. P-values for model terms are denoted as follows: n.s. ($p > 0.1$), . ($0.05 < p \leq 0.1$), * ($0.01 < p \leq 0.05$), ** ($0.001 < p \leq 0.01$), and *** ($p \leq 0.001$). L and Q denote the linear and quadratic terms, respectively.

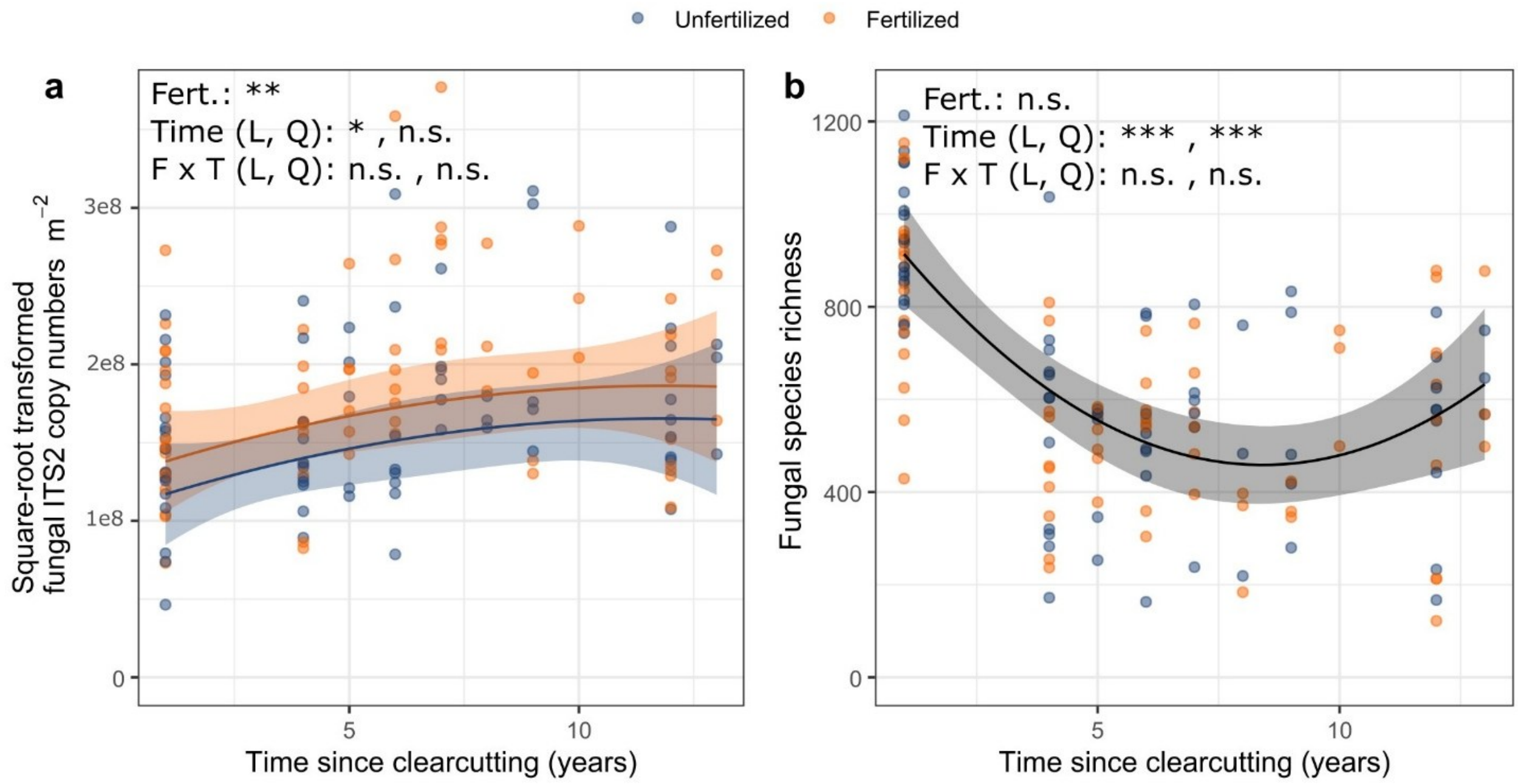

Fig. S3 Total fungal ITS2 copy numbers (a) and fungal species richness (b) in relation to time since clearcutting. Points indicate plot data and are coloured according to whether they come from clearcuts of previously fertilized (orange) or unfertilized (blue) forests. Predicted effects with their 95% confidence intervals from the linear mixed models for variables that had a significant effect are plotted on top of the raw data points. P-values for model terms are denoted as follows: n.s. ($p > 0.1$), . ($0.05 < p \leq 0.1$), * ($0.01 < p \leq 0.05$), ** ($0.001 < p \leq 0.01$), and *** ($p \leq 0.001$). L and Q denote the linear and quadratic terms, respectively.

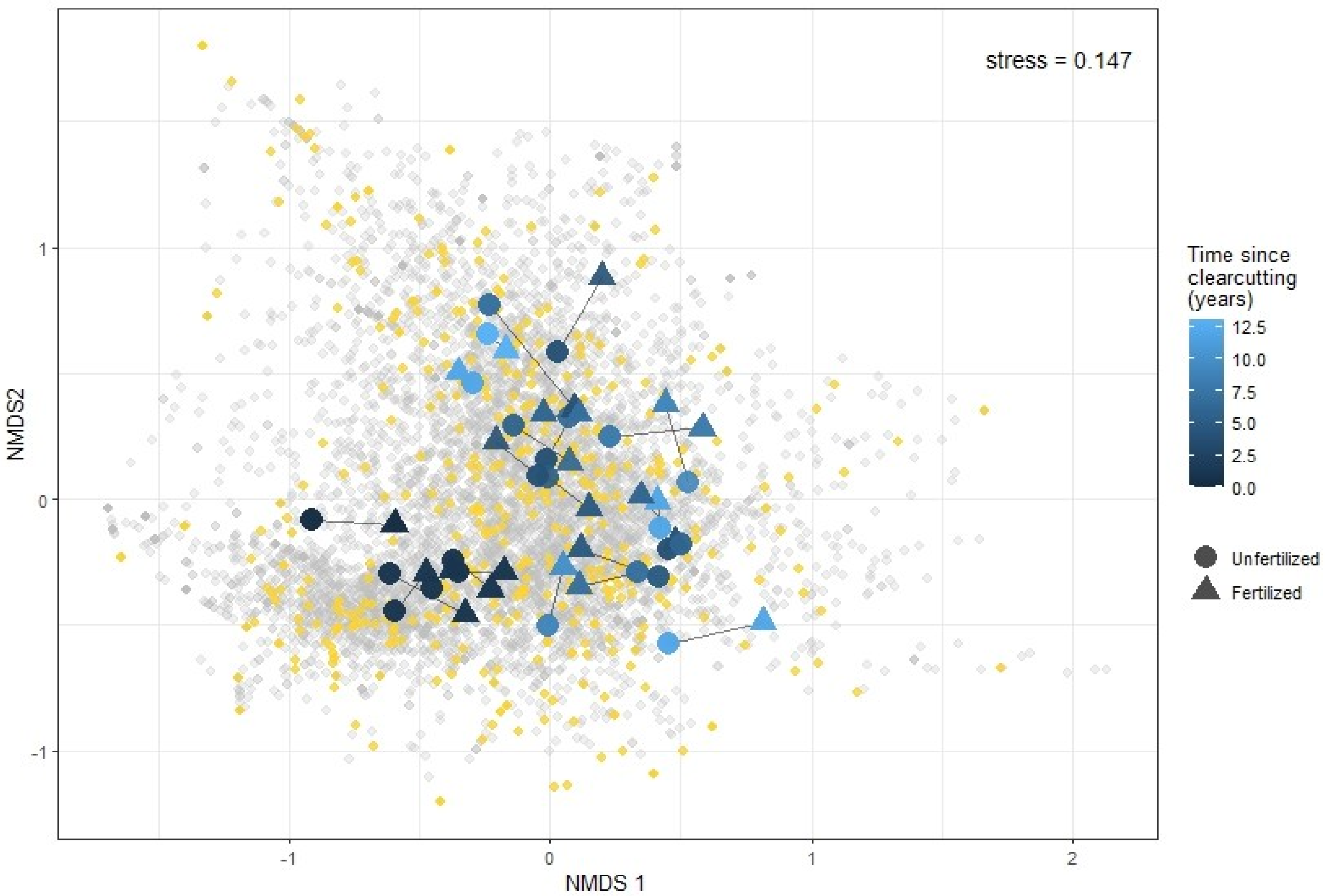

Fig. S4 NMDS of the total fungal communities. Large circles and triangles indicate the centroids per clearcut and paired clearcuts of previously fertilized (triangles) and unfertilized (circles) forests are connected with a line. Smaller dots indicate OTUs with an ectomycorrhizal lifestyle (yellow) or another or unknown lifestyle (grey).

3. Supplemental Tables

Table S1 Statistical significance of the coefficients from the linear mixed models on abundance and species richness of ectomycorrhizal and saprotrophic fungi and saprotrophic agaricomycetes. EcMF = ectomycorrhizal fungi; SapF = saprotrophic fungi; SapAg = saprotrophic agaricomycetes

**Variable**	**Fertilization effect**			**Time since clearcutting effect**						**Fertilization x time since clearcutting interaction**					
				**Linear term**			**Quadratic term**			**Linear term**			**Quadratic term**		
	***t*-value**	***DenDF***	***p*-value**	***t*-value**	***DenDF***	***p*-value**	***t*-value**	***DenDF***	***p*-value**	***t*-value**	***DenDF***	***p*-value**	***t*-value**	***DenDF***	***p*-value**
Abundance EcMF	0.9	21	0.402	**5.6**	**88**	**< 0.001**	1.8	88	0.075	-0.7	21	0.495	-1.4	21	0.164
Species richness EcMF	-1.3	21	0.203	**-2.2**	**88**	**0.028**	**5.6**	**88**	**< 0.001**	-0.6	21	0.533	-0.9	21	0.399
Abundance SapF	**3.0**	**21**	**0.007**	1.1	88	0.263	**-1.6**	**88**	**0.119**	0.3	21	0.757	-1.2	21	0.245
Species richness SapF	-1.1	21	0.281	**-3.4**	**88**	**0.001**	**3.2**	**88**	**0.002**	0.9	21	0.353	-0.4	21	0.695
Abundance SapAg	**2.2**	**21**	**0.038**	0.8	88	0.443	**-2.4**	**88**	**0.018**	1.3	21	0.196	**-2.3**	**21**	**0.034**
Species richness SapAg	-0.9	21	0.369	**-2.6**	**88**	**0.013**	**2.2**	**88**	**0.029**	1.3	21	0.202	-0.8	21	0.410

Table S2 Statistical significance of the coefficients from the linear mixed models per ectomycorrhizal fungal genus (for the ten most abundant genera), with square root transformed ITS2 copy numbers as response variable and fertilization, linear and quadratic term of time since clearcutting and the interaction of both as explanatory variables.

**Genus**	**Fertilization effect**			**Time since clearcutting effect**						**Fertilization x time since clearcutting interaction**					
				**Linear term**			**Quadratic term**			**Linear term**			**Quadratic term**		
	***t*-value**	***DenDF***	***p*-value**	***t*-value**	***DenDF***	***p*-value**	***t*-value**	***DenDF***	***p*-value**	***t*-value**	***DenDF***	***p*-value**	***t*-value**	***DenDF***	***p*-value**
Piloderma	-0.3	21	0.756	**8.5**	**88**	**< 0.001**	**5.9**	**88**	**< 0.001**	**-2.6**	**21**	**0.016**	**-2.2**	**21**	**0.038**
Suillus	0.8	21	0.432	**2.2**	**88**	**0.029**	-0.8	88	0.444	0.761	21	0.455	0.5	21	0.637
Thelephora	0.6	21	0.586	**3.8**	**88**	**< 0.001**	**-2.2**	**88**	**0.029**	0.1	21	0.951	-0.2	21	0.831
Russula	1.1	21	0.266	0.4	88	0.691	**2.6**	**88**	**0.011**	-0.2	21	0.806	-0.6	21	0.540
Cenococcum	0.2	21	0.814	**2.1**	**88**	**0.040**	**3.9**	**88**	**< 0.001**	-0.8	21	0.414	-1.8	21	0.085
Cortinarius	-0.6	21	0.574	**3.3**	**88**	**0.001**	**4.6**	**88**	**< 0.001**	-1.5	21	0.150	-0.3	21	0.739
Leccinum	1.3	21	0.201	**3.8**	**88**	**< 0.001**	1.6	88	0.111	-0.2	21	0.875	-1.7	21	0.102
Lactarius	0.8	21	0.442	**2.7**	**88**	**0.008**	1.8	88	0.079	0.7	21	0.499	0.3	21	0.739
Paxillus	-1.4	21	0.180	1.5	88	0.142	**-3.4**	**88**	**0.001**	0.3	21	0.758	1.5	21	0.139
Amanita	1.5	21	0.154	0.3	88	0.770	0.9	88	0.388	1.0	21	0.309	-0.9	21	0.391

Table S3 Significance of the coefficients of the linear mixed models, with square root transformed ITS2 copy numbers as response variable for the ten most abundant saprotrophic fungal genera. Due to their relatedness, similar ecology and similar model outcomes, *Trechispora* and *Luellia* have been merged at the family level, Hydnodontaceae (no other genera belonging to that family were present in the data). The full model included fertilization, linear and quadratic term of time since clearcutting and the interaction of both.

**Genus**	**Fertilization effect**			**Time since clearcutting effect**						**Fertilization x time since clearcutting interaction**					
				**Linear term**			**Quadratic term**			**Linear term**			**Quadratic term**		
	***t*-value**	***DenDF***	***p*-value**	***t*-value**	***DenDF***	***p*-value**	***t*-value**	***DenDF***	***p*-value**	***t*-value**	***DenDF***	***p*-value**	***t*-value**	***DenDF***	***p*-value**
Archaeorhizomyces	2.0	21	0.064	1.8	88	0.081	-1.4	88	0.158	-0.3	21	0.741	-0.4	21	0.693
Penicillium	**2.1**	**21**	**0.047**	0.6	88	0.574	-0.5	88	0.650	0.2	21	0.871	0.4	21	0.677
Mycena	1.4	21	0.167	0.3	88	0.775	**-2.3**	**88**	**0.025**	0.5	21	0.593	-0.8	21	0.417
Cladophialophora	-0.1	21	0.946	-0.5	88	0.652	-0.7	88	0.504	0.1	21	0.915	0.7	21	0.509
Mortierella	1.6	21	0.123	1.6	88	0.214	**-2.2**	**88**	**0.028**	0.1	21	0.916	0.5	21	0.638
Hydnodontaceae (*Luellia* and *Trechispora*)	**2.7**	**21**	**0.013**	1.3	88	0.195	**-2.7**	**88**	**0.009**	0.5	21	0.591	**-2.3**	**21**	**0.034**
Umbelopsis	1.4	21	0.180	0.2	88	0.833	**2.5**	**88**	**0.015**	1.7	21	0.100	-1.0	21	0.318
Phialocephala	1.4	21	0.184	-0.7	88	0.472	0.5	88	0.614	-0.1	21	0.919	-1.6	21	0.133
Solicoccozyma	1.8	21	0.087	0.8	88	0.452	-0.1	88	0.948	-0.3	21	0.740	1.2	21	0.238
